# Understanding decreased fertility in women carriers of the FMR1 premutation: a possible mechanism for Fragile X-Associated Primary Ovarian Insufficiency (FXPOI)

**DOI:** 10.1186/1742-4755-11-67

**Published:** 2014-08-19

**Authors:** Emmanuel Peprah

**Affiliations:** 1 National Institutes of Health, The Eunice Kennedy Shriver National Institute of Child Health and Human Development, 6100 Executive Blvd RM 5Z00, Rockville, MD 20852, USA

**Keywords:** FXPOI, Ovarian insufficiency, FMR1, CGG repeat, Female reproductive health

## Abstract

Fragile X syndrome (FXS) and its associated disorders are caused by the expansion of the CGG repeat in the 5′ untranslated region of the fragile X mental retardation 1 gene (*FMR1*). The full mutation, defined as >200 cytosine-guanine-guanine (CGG) triplet repeats, causes FXS. Individuals with 55–199 CGG repeats, classified as premutation carriers, are affected by two distinct disorders depending on their premutation status. Disorders associated with premutation carriers include: Fragile X-associated Tremor Ataxia Syndrome (FXTAS) and Fragile X-associated Primary Ovarian Insufficiency (FXPOI). The molecular similarities of FXTAS and FXPOI (e.g. overabundance of *FMR1* transcript and intranuclear inclusions) suggest that similar molecular mechanisms underlie both FXTAS and FXPOI. The current hypothesis describes the underlying mechanism for FXTAS as an mRNA gain-of-function mutation, however the underlying mechanism for FXPOI remains unresolved. New data suggests that repeat associated non-AUG (RAN) translation could underlie FXPOI.

## Background

Fragile X Syndrome (FXS; OMIM 300624) is caused by hypermethylation of the expanded CGG repeats adjacent to exon 1 of the Fragile X Mental Retardation 1 gene (*FMR1*); this mutation affects > 98% of individuals with FXS [1-3]. The disorder affects ~1/4000 males and ~1/8000 females in the general Caucasian population, with ~60% of individuals with the full mutation also having autism spectrum disorders [3-6]. The expanded CGG repeats can be categorized as common, intermediate, premutation, and full mutation alleles. Common alleles found in the general population usually contain 6–40 CGG repeats which are stable and usually do not expand upon transmission from parent to offspring. Intermediate alleles containing 41–60 CGG repeats have variable expansion risks whereas premutation alleles (i.e. 55–199 CGG repeats) are usually unmethylated and can expand to the full mutation (e.g. > 200 CGG repeats) upon transmission from parent to offspring [1,2]. In the full mutation, the expanded CGG repeat can be recognized as a CpG island resulting in methylation and transcriptional silencing of *FMR1*[1,2,7]. Transcription silencing of *FMR1* results in the loss of Fragile X Mental Retardation Protein (FMRP), which is necessary for neuronal development and cognition. FMR1 premutation carriers also have disorders associated with ovarian function including loss of fertility and hypoestrogenism (i.e., FXPOI) and neurodegeneration with associated memory loss and general Parkinsonism (i.e., FXTAS).

### FXPOI in female premutation carriers

Before the discovery of FXPOI, the consequences of being a female premature carrier was ascribed to the risk of passing on a full mutation to offspring; the ovarian dysfunction reported by many carriers was underappreciated. It was not until others observed that premature ovarian failure, defined as menopause before the age of 40, occurred in about 24% of *FMR1* premutation carriers compared to only ~1% of all women in the general population that the scientific community started to closely examine the association between FMR1 and ovarian function [8-12]. Reports indicate that premutation alleles with 59 to 99 CGG repeats are associated with an increased risk of ovarian dysfunction in female carriers [13,14]. This positive linear association of repeat size with ovarian dysfunction seems to plateau or decrease for premutation carriers with > 100 CGG repeats, however the exact threshold is currently unknown [15-19]. Furthermore, the linear association is not consistent because some intermediate repeats were associated with an increased risk for FXPOI compared to premutation repeats >100 [13,14]. Additionally, the length of the CGG repeat contributes to the variation observed in age of ovarian dysfunction resulting in a loss of reproductive capacity; currently the threshold for the onset of FXPOI is ambiguous [13,14,20]. Because of these differences between menopause and fragile x-associated ovarian insufficiency, the term "primary ovarian insufficiency" proposed by Welt and originally suggest by Albright compared to premature ovarian failure has been used to better describe the condition [21-23].

### Similarities of FXTAS and FXPOI

The molecular mechanism underlying FXPOI is enigmatic but using RAN translation in FXTAS as a model could illuminate the disease biology of FXPOI. Similar to FXTAS, oocytes in FMR1 premutation ovarian insufficiency mouse models display aberrant accumulation of protein, elevated levels of ubiquitination which reports suggest are consistent with evidence of the *FMR1* gain-of-function mechanism hypothesized in FXTAS [9,24]. Todd and colleagues suggest that inclusion formation in FXTAS could be mediated by the translational by-products of premutation alleles [25]. These aberrant FMRP by-products of non-AUG (RAN) translation, which have large glycine residues, mediate inclusion formation [25]. In fact, the mechanism was first reported by Zu and coworkers using spinocerebellar ataxia type 8 (SCA8) and myotonic dystrophy type 1 (DM1) as models to elucidate pathogenesis of triplet diseases [26]. Similar to other trinucleotide repeat expansion disorders (TNR), SCA8 and DM1 transcripts containing expanded cytosine-thymine-guanine (CTG) repeats were translated via RAN. The translational by-products of CTG repeats resulted in the accumulation of aberrant proteins with large leucine polypeptide tracks [26]. Further analysis revealed that these RAN translational by-products resulted in significant pathology including neurodegeneration, a hallmark of TNR. Moreover, Todd and colleagues demonstrated that similar to SCA8 and DM1, expanded CGG repeats (>90) were translated in Fragile X premutation carriers; the resulting translational products were high molecular weight FMRP with glycine residues, which the authors termed FMRpolyG [25]. FMRpolyG was shown to be important to inclusion formation and neurodegeneration, distinctive features of FXTAS and fully recapitulated the FXTAS phenotype [25].

### Conclusion and perspective

The lucidity of the molecular mechanism of FXTAS is emerging, however the pathobiology of FXPOI is opaque [27]. This would suggest that greater effort is needed to elucidate the underlying mechanisms of FXPOI via animal models and clinical studies using appropriate populations. Likewise, mice and other animal models could present opportunities to determine whether there are threshold effects for CGG repeat length in FXPOI and infer the biological mechanism. Namely, only a subset of premutation carriers develop FXPOI; one case report described premutation monozygotic twins discordant for the FXPOI phenotype although the X-inactivation ratios were similar for both sisters [28]. This is a very fascinating case report, and determining the genetic context of the premutation alleles by examining genetic elements (e.g., SNPS, CNVs) on the X chromosome (and if possible sequence their genomes) to determine the genetics associated with this is very compelling.

Finally, characterizing RAN translation by-products in animal models of FXPOI could provide the molecular evidence to understanding ovarian ageing associated with *FMR1*. Regardless, Todd and colleagues have shown that the RAN translation mechanism of premutation CGG repeats produces aberrant proteins essential for inclusion formation, resulting in FXTAS pathology. In FXPOI, RAN translation could occur in ovaries, granulosa cells, and/or other cell types. Given that inclusion formation has been found in several tissues of individuals with FXTAS, it is reasonable to assume that these inclusions exist in FXPOI. Confirmation of the importance of RAN translation by-products to FXPOI will significantly increase our understanding of the disorder and could provide an avenue to understanding ovarian dysfunction in FMR1 premutation carriers.

## Abbreviations

CNV: Copy number variation; FMR1: Fragile X mental retardation 1 gene (*FMR1*); FXTAS: Fragile X-associated tremor ataxia syndrome (FXTAS); FXPOI: Fragile X-associated primary ovarian insufficiency (FXPOI); RAN: Repeat associated non-AUG (RAN) translation; SNP: Single nucleotide polymorphisms; TNR: Trinucleotide repeat expansion disorders.

## Competing interests

The author declares he has no other competing interests.
